# Assessment of the bioactivity of bioinspired magnesium oxide nanoparticles from the *Azadirachta indica* extract

**DOI:** 10.3389/fbioe.2024.1480694

**Published:** 2024-11-29

**Authors:** Laila M. Al-Harbi, Mohammed Ezzeldien, Ahmed A. Elhenawy, Alaa Hassan Said

**Affiliations:** ^1^ Department of Chemistry, Faculty of Science, King Abdulaziz University, Jeddah, Saudi Arabia; ^2^ Physics Department, College of Science, Jouf University, Sakaka, Saudi Arabia; ^3^ Department of Chemistry, Faculty of Science, Al-Azhar University, Nasr CityCairo, Egypt; ^4^ Department of Chemistry, Faculty of Science, Al-Baha University, Al-Baha, Saudi Arabia; ^5^ Electronics and Nano Devices Lab, Faculty of Science, South Valley University, Qena, Egypt

**Keywords:** *Azadirachta indica*, neem, green magnesium oxide nanoparticles, antidiabetic, anti-inflammatory, anticancer, antibacterial

## Abstract

*Azadirachta indica* (neem) extract was used to biologically synthesize magnesium oxide nanoparticles (MgO NPs). The synthesized NPs were characterized using X-ray diffraction (XRD), thermogravimetric analysis (TGA), transmission electron microscopy (TEM), scanning electron microscopy (SEM), Fourier-transform infrared (FTIR), and UV-vis spectroscopy. Antioxidant, anticancer, antibacterial, antidiabetic, and anti-inflammatory activities were analyzed for the synthesized MgO NPs and neem extract. The obtained results confirmed the synthesis of spherical magnesium oxide nanoparticles with an average particle size of 23 nm using XRD. The samples exhibited good thermal stability and high stability in biological media. Compared to the neem extract and chemically synthesized magnesium oxide nanoparticles, the bioinspired magnesium oxide nanoparticles showed considerable free radical scavenging activity, with an IC_50_ value of 69.03 μg/mL. In addition, they reflected high selectivity to liver hepatic cancer cells with an IC_50_ value of 94.85 μg/mL without inducing any damage to human umbilical vein endothelial cells. The antibacterial activity of the bioinspired magnesium oxide nanoparticles demonstrated comparable effectiveness in treating both Gram-positive and Gram-negative bacterial strains. Furthermore, the produced bioinspired magnesium oxide nanoparticles showed a high percentage of inhibition for both α-amylase and α-glucosidase enzymes with an IC_50_ value of 61. 53 and 50.6 μg/mL, respectively. In addition, the bioinspired magnesium oxide nanoparticles also showed a higher denaturation inhibition percentage with an IC_50_ value of 6.66 μg/mL, indicating strong anti-inflammatory action. These enhanced abilities usher in a new bioinspired magnesium oxide nanoparticle bio-application era. Consequently, further *in vivo* studies are needed to assess the kinetic properties of these nanoparticles.

## Graphical Abstract

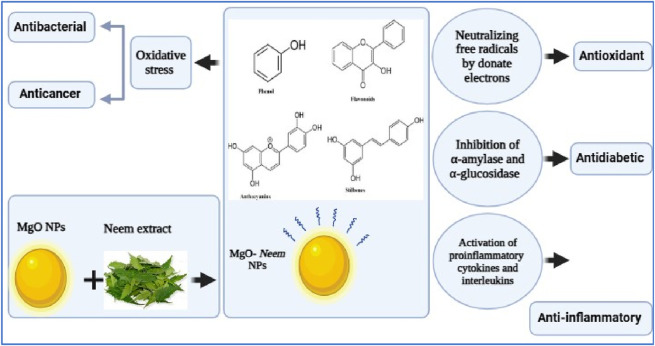

## 1 Introduction

Bioinspired metal oxide nanoparticles (NPs), such as magnesium oxide, have gained global attention due to their outstanding physiochemical characteristics and bioactivities. The biological synthesis of NPs can readily mimic these features to make ecologically clean NPs with better bioactivities (antioxidant, anticancer, antibacterial, antidiabetic, and anti-inflammatory). Magnesium oxide nanoparticles (MgO NPs) are eco-friendly and commercially viable. They are essential in industrial applications due to their high refractive index, excellent corrosion resistance, strong thermal conductivity, lower electrical conductivity, and high biocompatibility. These unique physicochemical properties make them significant in various fields (Thakur et al., 2022; John et al., 2016; Pilarska et al., 2017). They are used in photochemical products to enhance light absorption and conversion. In electronics, MgO NPs improve materials used in capacitors and insulators. In ceramics, they enhance mechanical strength and thermal resistance.

Additionally, they serve as effective catalysts in various chemical processes and are explored in pharmaceutical development for drug delivery and therapeutic applications. Their diverse usage highlights their importance in advancing technology across multiple industries (Singh et al., 2020; Abdulkhaleq et al., 2020; Sharma et al., 2020). In addition to being important in the bioremediation of contaminants, MgO NPs have been retained in paints, catalysts, refractory additives, and superconducting products (Singh et al., 2020; Abdulkhaleq et al., 2020; Kant et al., 2021; Mantilaka et al., 2018; Gajengi et al., 2017). MgO NPs show great promise in medicine. They can treat heartburn, promote bone regrowth, and act as antimicrobial and antitumor agents. Their diverse applications power their potential to enhance medical treatments (Sharmila et al., 2019; Abdullah and Mohammed, 2021; Bindhu et al., 2016).

The growing use of NPs in various fields has recently led to several challenges. There are increasing concerns about environmental contamination, bacterial multidrug resistance, depletion of natural energy resources, and healthcare-related issues. These challenges have heightened interest in designing and developing environmentally conscious products to address these pressing issues (Kumar et al., 2023).

Green synthesis is increasingly preferred over traditional methods due to its key advantages: simplicity, cost-effectiveness, and safety. It uses non-toxic, eco-friendly reagents, reducing hazardous waste and minimizing the environmental impact. Additionally, the scalability of green synthesis allows for efficient production suitable for commercial applications, leading to an increase in bioinspired NP production, which leverages natural processes and materials for unique properties. A broader shift toward sustainable practices in nanotechnology offers innovative solutions while promoting environmental responsibility (Dadkhah and Tulliani, 2022; Soltys et al., 2021; Aigbe and Osibote, 2024). A biogenic solvent is needed for bioinspired NP synthesis to facilitate the reduction process and stabilize the resultant NPs. Plants, fungi, and microorganisms have been investigated as reducing and capping agents for NP synthesis (Jeevanandam et al., 2022; Srivastava et al., 2015; Yuliarto et al., 2019; Ashour et al., 2023). Metal ions are reduced by the action of plant and bacterial biomolecules such as amines, carbohydrates, ketones, amino acids, phenols, aldehydes, proteins, and carboxylic acids (Siddiqi and Husen, 2016; Kumar et al., 2021). Compared to bacteria and/or fungus-mediated synthesis, using the plant extract to produce NPs at scale is a straightforward process (Singh et al., 2018). Due to the dominance of biomolecules in different parts of plants with high concentrations, the biosynthesis of NPs was reported using plant leaves, roots, seeds, and fruits (Jadoun et al., 2021).

The biosynthesis of MgO NPs was reported using various types of plants such as *Solanum trilobatum* (Narendhran et al., 2019), *Rosmarinus officinalis* (Abdallah et al., 2019), *Matricaria chamomilla* L. (Ogunyemi et al., 2019), *Calotropis gigantea* (Hii et al., 2018), *Moringa oleifera* (Vijayakumar et al., 2023), *Limonia acidissima* (Nijalingappa et al., 2019), and *Azadirachta indica* (Moorthy et al., 2015; Aravind Kumar et al., 2019). The bioinspired MgO NPs showed comparable antioxidant, anticancer, antibacterial, antidiabetic, and anti-inflammatory activities.

*A. indica* (Neem) is a familiar medical plant grown in tropical and subtropical climates. As a member of the Meliaceae family, it contains many constituents, such as nimbin, nimbidin, nimbolide, and limonoids. Phenolic and flavonoid phytochemicals are responsible for their antibacterial, antifungal, and antimicrobial properties (Ali et al., 2021; Seriana et al., 2021). Neem leaves, flowers, and seeds were reported to treat skin allergies, tooth infections, and wound healing (Alzohairy, 2016). Furthermore, the neem extract showed radiosensitization activity after irradiation with infrared light in neuroblastoma (NB), inhibiting the anti-apoptotic signaling cascade (Veeraraghavan et al., 2011). Recently, the neem extract was applied in the green synthesis of palladium (Amrutham et al., 2020), silver (Chand et al., 2019), zinc oxide (Saravanan et al., 2020), copper oxide (Nagar and Devra, 2018), titanium oxide (Thakur et al., 2019), and manganese oxide NPs (Moorthy et al., 2015; Aravind Kumar et al., 2019).

This research innovatively explores the impact of the neem extract on the structural, optical, and bioactive properties of MgO NPs through a bioinspired synthesis approach. By comparing two distinct synthesis methods—one utilizing sodium hydroxide as a reducing agent and the other incorporating the neem extract—this study aims to highlight the potential benefits of neem extracts in NP synthesis and bioactivities.

## 2 Experiment details

### 2.1 Materials

The following materials were used to synthesize MgO NPs and perform cell culture experiments without purification. Magnesium nitrate, Mg (NO_3_)_2_.6H_2_O, ethanol, propanol, acarbose, 3,5-dinitrosalicylic acid (DNSA), bovine serum albumin, diclofenac sodium, and dimethyl sulfoxide (DMSO) were purchased from Alfa Aesar, United States. Dulbecco’s modified Eagle’s medium, L-glutamine, and fetal bovine serum were purchased from Life Science Production, United Kingdom. Penicillin–streptomycin, phosphate-buffered saline (PBS), and trypsin–EDTA were purchased from Lonza, Germany. Furthermore, 3-[4,5-dimethylthiazol-2-yl]-2,5 diphenyltetrazolium (MTT), 2,2-diphenyl-1-picrylhydrazyl (DPPH), 2′,7′-di-chlorodihydrofluorescein diacetate acetyl ester (DCFH-DA), yeast α-glucosidase, p-nitrophenyl-α-D-glucopyranoside (pNPG), and α-amylase were purchased from SERVA Electrophoresis GmbH, Heidelberg, Germany. Liver hepatocellular cells (HepG2 cells) were purchased from the Egyptian holding company for biological products and vaccines (Vacsera), Giza, Egypt. The following bacterial strains were donated by the Department of Botany and Microbiology, Faculty of Science, South Valley University, Qena, Egypt: *Salmonella typhimurium* (ATCC 14028), *B. subtilis* (ATCC 29213), *E. coli* (ATCC 25922), and *Staphylococcus aureus* (ATCC 29213). This work was approved by the Ethics Committee of South Valley University, Faculty of Science (Permit Number: 001/08/24).

### 2.2 Aqueous extract of neem

Neem leaves were collected from a local farm, South Valley University Farm, washed thoroughly with distilled water, and left to dry for 24 h. Then, 50 g of the dried leaves were cut and boiled in 200 mL of distilled water for 30 min. The final extract was filtered three times using Whatman paper number 1 and stored at 4°C until further use.

### 2.3 Synthesis of MgO NPs

**FIGURE 1:**
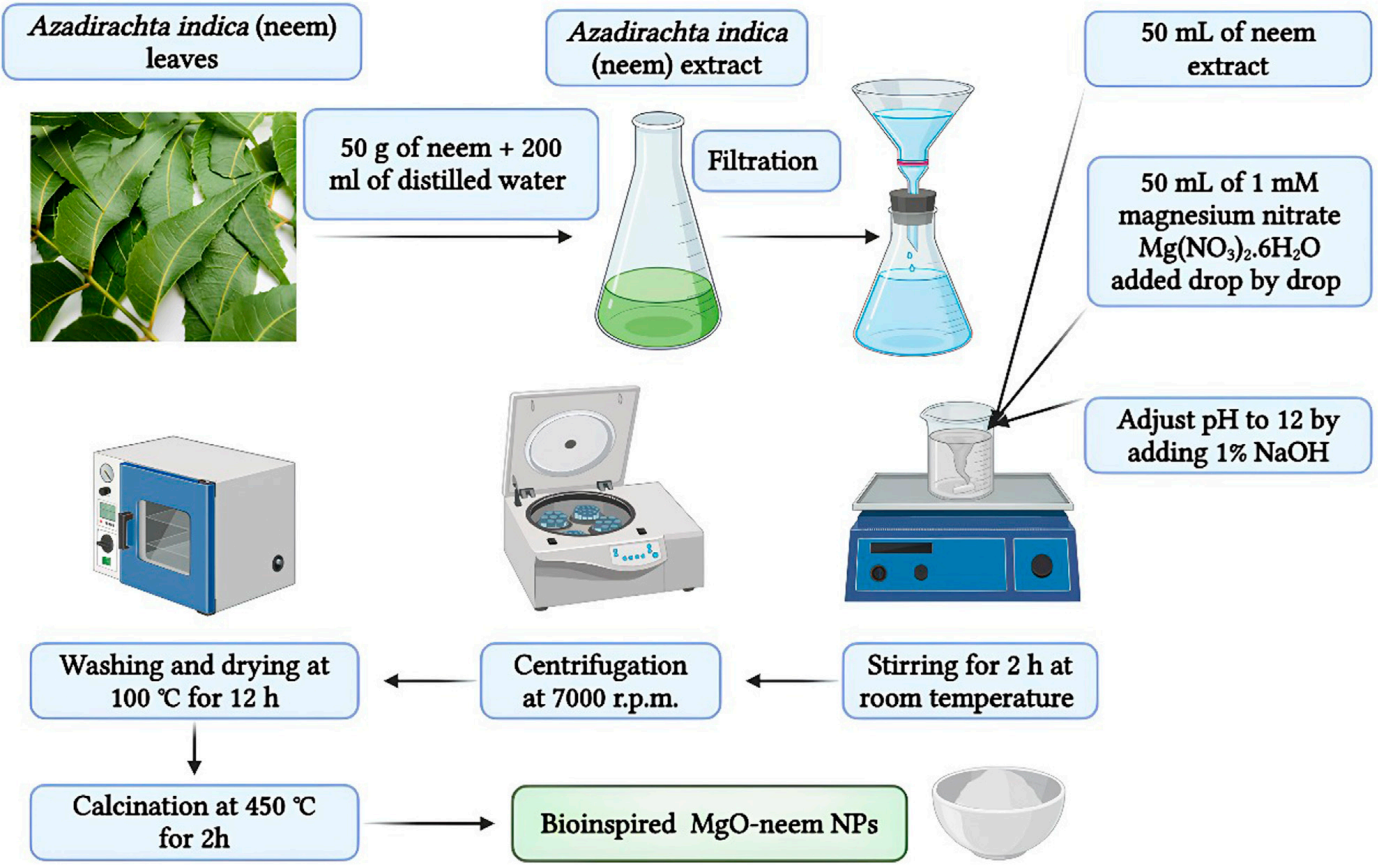
Schematic illustration of the synthesis conditions of chemically synthesized and bioinspired MgO NPs.

Two MgO NP samples, bioinspired MgO (denoted as MgO–neem NPs) and chemical MgO (denoted as MgO NPs), were prepared by the co-precipitation technique with magnesium nitrate serving as the magnesium precursor (Moorthy et al., 2015; Umaralikhan and Jamal, 2018; Vijayakumar et al., 2021). The magnesium reduction process occurs with neem extract and sodium hydroxide in the two samples, respectively. In brief, 50 mL of 1 mM magnesium nitrate solution was added drop by drop to 50 mL of the reducing agent under stirring at 600 rpm for 30 min. The pH of the solution was adjusted to 12 by adding 1% NaOH. To maximize the formation of the Mg (OH)_2_ precipitate, the aging procedure was carried out by stirring both samples for 2 h at room temperature. Following a 24-h precipitation period at room temperature, the generated NPs were recovered by centrifugation at 7,000 rpm, followed by three rounds of washing to eliminate unreacted ions. Lastly, the samples were dried in an oven at 100°C for 12 h and then calcinated for 2 h at 450°C (Figure 1).

### 2.4 Characterization of MgO NPs

An X'Pert PRO-PAN X-ray Machine with Cu-Kα radiation at 40 kV and 30 mA, high-resolution transmission electron microscope (JEOL, model JEM 2100, Japan), and scanning electron microscope (JEOL SEM model JSM-5500, Japan) were used to explore the structural and morphological future of MgO NPs. Spectroscopic analysis was conducted using a Fourier transformation infrared (model 6100, Jasco) spectrometer and UV–visible spectrophotometer (model SPECORD 200 PLUS, Analytik Jena, Germany). Thermal stability was assessed using a Shimadzu (TGA-50H) instrument at a heating rate of 20^o^C/min under a constant flowing nitrogen atmosphere.

### 2.5 Bioactivity of MgO NPs

Different bioactivities of MgO, MgO–neem NPs, and neem extract were investigated. Table 1 summarizes the experimental details of each one. For each spectroscopic measurement, the absorption was detected using a UV–visible spectrophotometer according to each assay condition for standard (A_C_) and test samples (A_S_). All experiments were performed in triplicates, and the calculated values were expressed as the mean (±SD). The IC_50_ value was obtained from the graph of the inhibition percentage against the sample concentration.

**TABLE 1:** Experimental details of the measured bioactivates used in this study.

	Activity	MgO NPs	MgO–neem NPs	Neem extract
Measured parameter	Antioxidant activity	$\text{FRSC }\left(\%\right)=\left(\frac{A_{C517}-A_{S517}\;}{A_{C517}}\right)\times 100$ (1)		
Mechanism of action		Neutralizing free radical		
Used concentrations (µg/mL)		1,000, 500, 250, 125, 62.5, 31.25, 15.625, and 7. 8		
Bioactive medium		2,2-Diphenyl-1-picrylhydrazyl (DPPH)		
Standard material		Ascorbic acid		
Protocol reference		Alghamdi et al. (2023)		
Measured parameter	Antibacterial activity	Zone of inhibition (mm)		
Mechanism of action		Inducing oxidative stress		
Used concentrations (µg/mL)		25, 50, and 100		20
Bioactive medium		• *S. aureus* (ATCC 29213) • *B. subtilis* (ATCC 29213) • *E. coli* (ATCC 25922) • *S. typhimurium* (ATCC 14028)		
Standard material		Gentamycin with a concentration of 20 μg/mL		
Protocol reference		Balouiri et al. (2016)		
Measured parameter	Anticancer activity	$\text{Cell viability}\%=\frac{A_{C590}-A_{S590}}{A_{C590}}$ (2)		
Mechanism of action		Inducing oxidative stress		
Used concentrations (µg/mL)		1,000, 500, 250, 125, 62.5, 31.25, 15.8, and 7.8		
Bioactive medium		• Liver hepatic cancer cells (HepG2) • Human umbilical vein endothelial cells (HUVECs)		
Standard material		Doxorubicin		
Protocol reference		Kamal et al. (2022)		
Measured parameter	Oxidative stress	Fluorescence intensity at λ_EX_/λ_EM_ = 485 nm/535 nm		
Mechanism of action		Oxidization of non-fluorescent 2′,7′-di-chlorodihydrofluorescein diacetate acetyl ester (DCFH-DA) to fluorescent dichlorofluorescein (DCF) (λ_EX_/λ_EM_ = 485 nm/535 nm)		
Used concentrations (µg/mL)		1,000, 500, 250, 125, 100, and 50		
Bioactive medium		• HepG2 • HUVECs		
Standard material		-		
Protocol reference		Amin et al. (2023)		
Measured parameter	Antidiabetic activity	• $\alpha -glucosidase\;inhibition\;\%=\frac{A_{C\left(405\right)}-A_{S\left(405\right)}}{A_{C\left(405\right)}}\times 100$ (3) • $\alpha -\text{amylase }inhibition\;\%=\frac{A_{C\left(540\right)}-A_{S\left(540\right)}}{A_{C\left(540\right)}}\times 100$ (4)		
Mechanism of action		• Inhibition of α-glucosidase • Inhibition of α-amylase		
Used concentrations (µg/mL)		1,000, 500, 250, 125, 62.5, 31.25, 15.63, 7.81, 3.91, and 1.95		
Bioactive medium		• Yeast α-glucosidase and p-nitrophenyl-α-D-glucopyranoside (pNPG) • 3,5-Dinitrosalicylic acid (DNSA)		
Standard material		Acarbose		
Protocol reference		(Kim et al., 2004; Wickramaratne et al., 2016)		
Measured parameter	Anti-inflammatory activity	$\text{Inhibition }\%=\frac{A_{C\left(660\right)}-A_{S\left(660\right)}}{A_{C\left(660\right)}}\times 100$ (5)		
Mechanism of action		Activation of pro-inflammatory cytokines and interleukins, stabilizing protein structures and preventing denaturation		
Used concentrations (µg/mL)		1,000, 500, 250, 125, 62.5, 31.25, 15.6, 7.8, 3.9, 2, 1, and 0.5		
Bioactive medium		Bovine serum albumin (BSA)		
Standard material		Diclofenac sodium		
Protocol reference		Fahaduddin (2024)		

#### 2.5.1 Antioxidant activity (DPPH assay)

The free radical scavenging (FRSC) activity was evaluated using a standardized DPPH assay (Alghamdi et al., 2023). Test samples and standard material were dissolved in methanol to prepare a series of concentrations, as shown in Table 1. Subsequently, 3 mL of DPPH was added to each test tube and kept in the dark at room temperature for 30 min. Ultimately, the absorbance (A_517_) of the test samples and the standard was detected spectrophotometrically at 517 nm, and FRSC activity against DPPH was evaluated using Equation 1 (Table 1).

#### 2.5.2 Antibacterial activity

The capacity of the test samples to induce bacterial cell death was evaluated using the agar diffusion disk method (Balouiri et al., 2016). Luria-Bertani (LB) broth medium was used to grow the four bacterial strains listed in Table 1 and acquire the bacterial suspensions. The next day, hygienic disks with a 6-mm diameter containing test samples and standard material with the concentrations listed in Table 1 were used to cover the cultured bacterial plates, which were then incubated at 37°C for 24 h. The zone of inhibition was then measured in millimeters.

#### 2.5.3 Anticancer activity

The cytotoxicity of test samples and standard material against two cell lines, listed in Table 1, was screened using the MTT assay (Kamal et al., 2022). Cells were seeded with a density of 10,000 cells per well in a 96-well plate in complete DMEM supplemented with 10% fetal bovine serum (FBS) and 1% penicillin/streptomycin and incubated for 24 h at 37°C with 5% CO_2_. The next day, after the cells reached a confluency of 80%, detached cells were removed by washing the plates with PBS, and 100 μL of the new culture medium containing test samples and standard with the concentrations listed in Table 1 was added to each well under incubation conditions (at 37°C with 5% CO_2_). The exposure time was set to be 24 h; after this period, the cells were washed again with PBS, and 80 µL of FBS-free medium mixed with 20 µL of MTT reagent was added to each well under incubation conditions (at 37°C with 5% CO_2_). After 3 h, 100 µL of stooping regent DMSO was added to each well to halt the reaction, and the mixture was left under shaking in the dark for 15 min. Finally, the absorbance at 590 nm was measured, and cell viability was evaluated using Equation 2 given in Table 1.

#### 2.5.4 Oxidative stress assay

Oxidative stress (ROS) of the MgO NPs was evaluated using the DCFH-DA assay (Amin et al., 2023). Generally, the generated ROS oxidizes the non-fluorescent DCFH-DA into the brightly fluorescent compound dichlorofluorescein (DCF) (λ_EX_/λ_EM_ = 485 nm/535 nm). This assay was performed in both HepG2 and HUVEC cell lines under the same culturing conditions as the MTT assay. Cells were exposed to test samples at the concentrations mentioned in Table 1 for 24 h. The cells were then washed with PBS and incubated with 100 µL of the fresh culture medium containing 80 µL of the serum-free medium and 20 µL of the DCFH-DA reagent under incubation conditions (at 37°C with 5% CO_2_) for half an hour in the dark. After treatment, the cells were washed with PBS, and the microplate reader detected the fluorescence intensity at λ_EX_/λ_EM_ = 485 nm/535 nm.

#### 2.5.5 Antidiabetic activity

##### 2.5.5.1 α-Glucosidase inhibition assay

The ability of MgO NPs to inhibit the activity of the α-glucosidase enzyme was screened using yeast α-glucosidase and p-nitrophenyl-α-D-glucopyranoside (pNPG) (Kim et al., 2004). The tested samples and standards were prepared in PBS at the concentrations mentioned in Table 1. The concentrations of α-glucosidase and pNPG were adjusted to 0.1 M of PBS (1U/mL) and 10 mM, respectively. A measure of 100 μL of the prepared α-glucosidase was added to each test tube and kept at 37°C for 20 min. Subsequently, 10 μL of the prepared pNPG was added to each test tube and incubated at 37°C for 30 min. Finally, 650 μL of the stopping reagent (sodium carbonate, 1 M) was added to halt the reaction. The absorbance at 405 nm was recorded, and the inhibition percentage was evaluated using Equation 3 (Table 1).

##### 2.5.5.2 α-Amylase inhibitory

The α-amylase inhibition test was carried out using the DNSA technique (Wickramaratne et al., 2016). Tested samples and standards were prepared in PBS at the concentrations listed in Table 1. A measure of 10 μL of α-amylase (2 units/mL) was mixed with 20 μL of each sample in the test tube and kept at 37°C for 20 min. Then, 200 μL of 1% potato starch in PBS (100 mM) was added to each test tube and kept at 37°C for a further 30 min. The reaction was terminated by adding 100 μL of the DNSA reagent to each test tube and boiling at 90°C for 10 min. After cooling to room temperature, the absorbance was measured at 540 nm, and the inhibition percentage was evaluated using Equation 4 (Table 1).

#### 2.5.6 Anti-inflammatory activity

The anti-inflammatory activity of the sample was determined using a protein denaturation assay (Fahaduddin, 2024). Any material with anti-inflammatory properties could stabilize protein structures and prevent denaturation. The tested samples and standards were prepared in PBS at the concentrations mentioned in Table 1. A measure of 3 mL of 1% bovine serum albumin (BSA) was added to each tube and incubated in a water bath at 55°C for 20 min. The absorbance was detected at 660 nm, and the inhibitory percentage was evaluated using Equation 5 (Table 1).

### 2.6 Statistical analysis

Statistical variation among the obtained results was assessed using a one-way analysis of variance (ANOVA) with the Statistical Package for Social Sciences (SPSS). The results are expressed as the mean ± standard deviation, and *p* < 0.05 was considered statistically significant.

## 3 Results and discussion

### 3.1 Characterization of MgO NPs

#### 3.1.1 Structural analysis using X-ray diffraction

**FIGURE 2:**
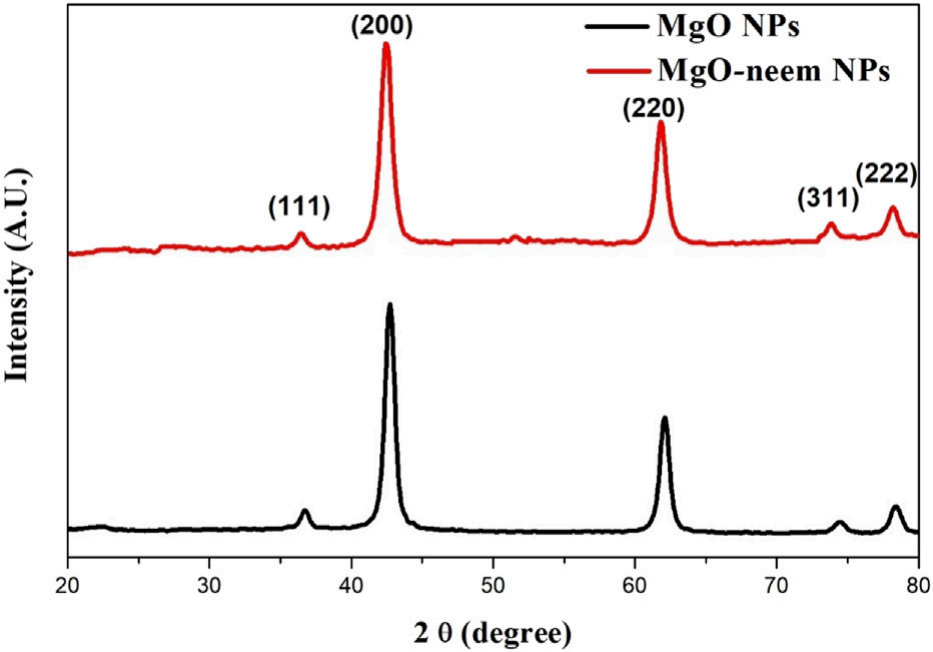
XRD pattern of MgO and MgO–neem NPs.

The successful formation of both MgO NPs and MgO–neem NPs was confirmed by the presence of distinct fingerprint diffraction peaks characteristic of MgO NPs, as illustrated in Figure 2. These peaks indicate the crystalline nature and structural integrity of the synthesized NPs, confirming that the synthesis methods used effectively produced high-quality MgO NPs. Five diffraction peaks appeared in both samples at 2θ = 36.7, 42.76, 62.08, and 78.4 for MgO NPs, which shifted to lower 2θ = 36.46, 42.4, 61.84, 73.84, and 78.22 for MgO–neem NPs, respectively. These diffraction peaks belonged to (111), (200), (220), (311), and (222) diffraction plans, respectively. The detected diffraction peaks were assigned to cubic face-centered crystal (FCC) structure MgO NPs, which is in line with the reported card (JCPDS No. 87-0652) (Wang et al., 2024).

The cubic FCC structure is characterized by lattice parameters (a = b = c = 4.215787 A°). The lattice parameter for both samples was calculated using Equation 6 (Lee et al., 2016):

$$d_{hkl}=\frac{a}{\sqrt{h^{2}+k^{2}+l^{2}}}.$$ (6)

The average crystal size was calculated using Scherer’s Equation 7 (Kamal et al., 2022):

$$D=\frac{0.9\lambda }{\Gamma \mathrm{Cos}\theta },$$ (7)

where D is the mean crystal size, λ is the X-ray wavelength source, 0.9 is constant for crystal shape, θ is the diffraction angle, and Γ is the full-width at half-maximum of the diffraction peak. The dislocation density (δ, nm^-2^) was calculated as 1/D^2^.

A slight increase was observed in the crystal size of bioinspired MgO–neem NPs compared with MgO NPs from 21.807 ± 2.053 to 23.09 ± 2.78 (nm) due to the coating action of biomolecules in the neem extract. This increase was combined with an enlargement in the lattice parameter *a* from 4.225 to 4.248 (A°) and an increase in the dislocation density (see Table 2). The results establish that neem extracts are reducing agents for synthesizing MgO NPs without distorting the crystal structure. Similar results were reported for the green synthesis of MgO NPs from different biological sources (Moorthy et al., 2015; Aravind Kumar et al., 2019).

**TABLE 2:** Crystallographic data of MgO NPs as analyzed from XRD, the full-width at half-maximum was calculated using OriginLab software, the crystal size (D, nm) was calculated using Scherer’s equation, and dislocation density (δ, nm^2^) was calculated as 1/D^2^.

Sample	MgO NPs					
Plane	2θ	d_hkl_	Γ	D (nm)	δ * 10^−4^ (nm^−2^)	*a = b = c*
***111***	36.7	2.445	0.463	18.873	28.073	4.236
***200***	42.76	2.112	0.385	23.135	18.683	4.224
***220***	62.08	1.493	0.403	24.020	17.331	4.223
***311***	74.44	1.272	0.504	20.665	23.414	4.222
***222***	78.4	1.218	0.479	22.344	20.029	4.220
Average (nm)				21.807	21.506	4.225
STDV				2.053	4.311	0.006

Sample	MgO–neem NPs					
Plane	2θ	d_hkl_	Γ	D (nm)	δ * 10^−^^4^ (nm ^-2^)	*a = b = c*
***111***	36.46	2.461	0.426	20.498	23.798	4.263
***200***	42.4	2.129	0.345	25.786	15.039	4.258
***220***	61.84	1.498	0.397	24.352	16.861	4.238
***311***	73.84	1.281	0.526	19.723	25.706	4.251
***222***	78.22	1.220	0.426	25.092	15.882	4.228
Average (nm)				**23.090**	**19.457**	**4.2480**
STDV				**2.780**	**4.922**	**0.014**

Bold values represent the average ± SD for the measured parameters.

#### 3.1.2 Electron microscope technique

**FIGURE 3:**
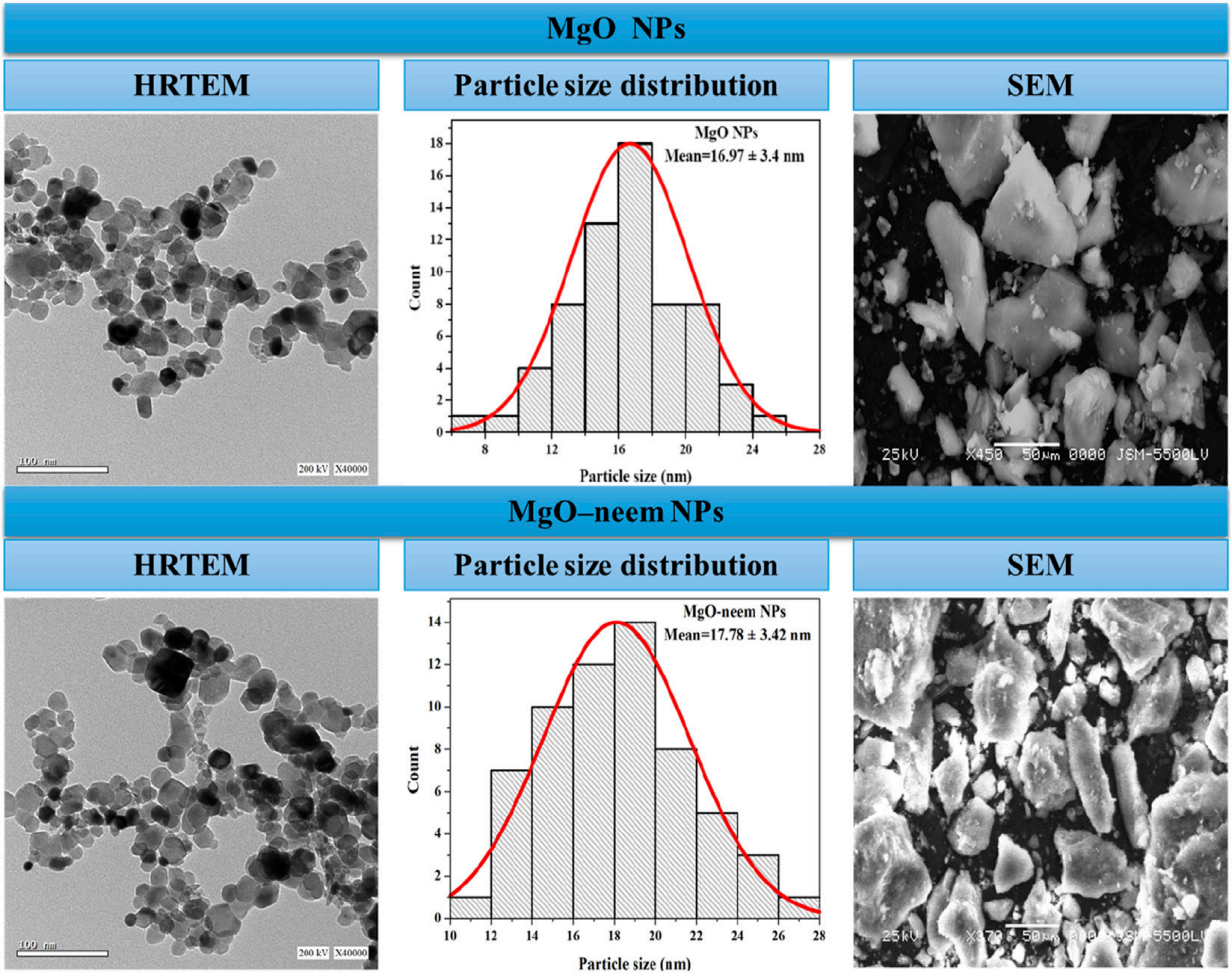
HRTEM and SEM images of MgO and bioinspired MgO–neem MgO NPs. HRTEM images with a scale of 100 nm were recorded at 200 KV, while SEM images with a scale of 50 µm were recorded at 25 KV. Particle size distribution was calculated using ImageJ software (n = 100).

The high-resolution transmission electron microscope (HRTEM) technique was used to study the structural future of NPs in terms of particle size and shape. The formed MgO NPs showed semispherical shapes (Figure 3). In HRTEM images, some aggregations of MgO NPs and MgO–neem NPs were observed. These aggregations were also observed in SEM images, mainly due to surface attractive interactions in the nanoscale. The green synthesis of NPs can contribute to this agglomeration by coating the MgO NP surface with biomolecules, which can interact with the surrounding molecules via many Coulombic interactions (Arabi et al., 2020; Shanavas et al., 2020). ImageJ software was used to calculate the particle size of MgO NPs. A total of 100 particles per image were selected, and then particle size distribution was plotted. The mean particle size of MgO NPs was 16.97 ± 3.4 nm, while the mean particle size of MgO–neem NPs was 17.78 ± 3.42 nm. These results were in line with the XRD data and reported results for bioinspired MgO NPs (Nijalingappa et al., 2019; Vijayakumar et al., 2021; Pachiyappan et al., 2020).

#### 3.1.3 UV–visible spectroscopy

**FIGURE 4:**
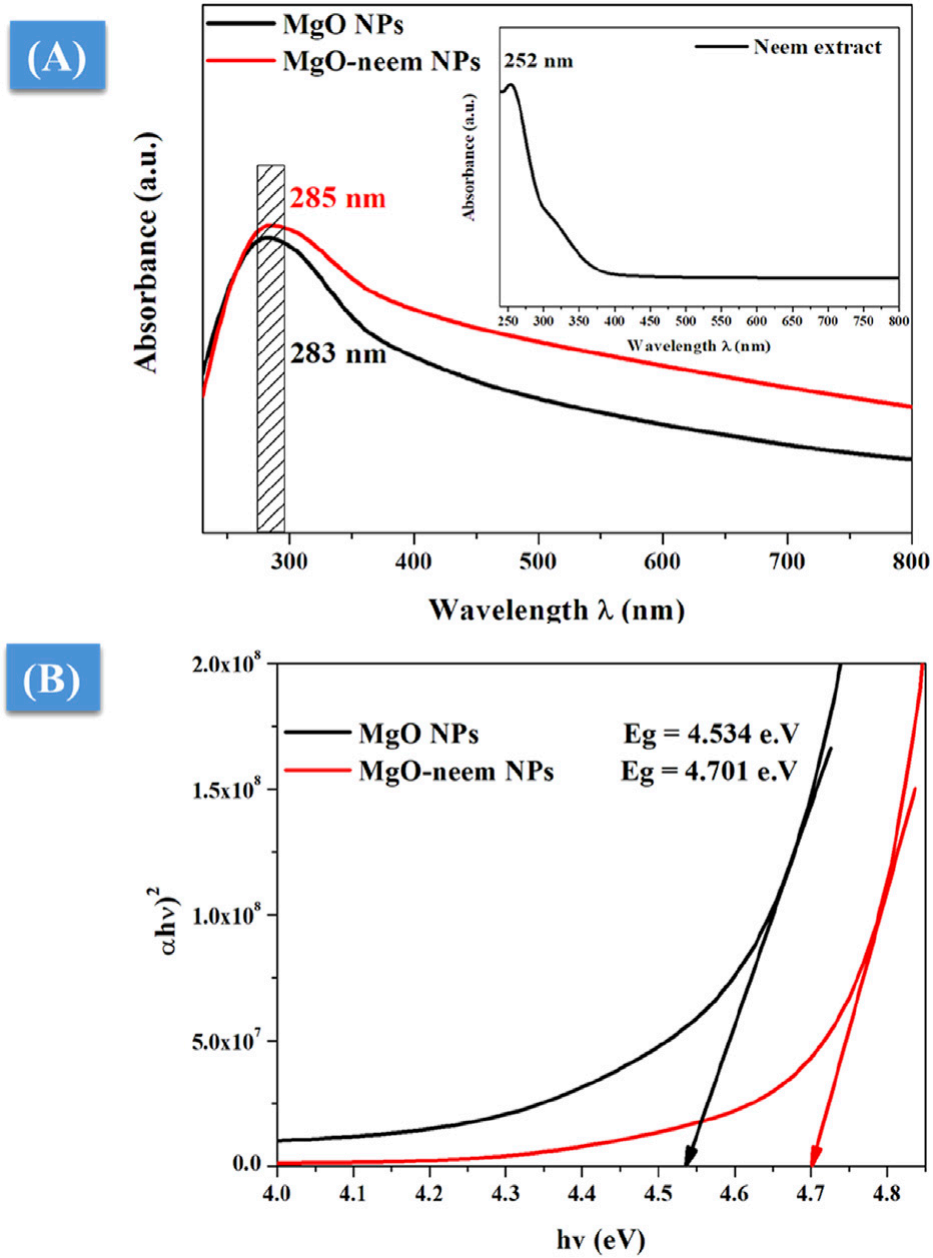
UV-vis spectroscopy of MgO and bioinspired MgO–neem NPs: **(A)** UV-vis absorption spectra and **(B)** optical band gap calculated using Tauc’s equation.

UV-vis spectrometry was used to characterize the photocatalytic activity of NPs (Devanand et al., 2013). The absorption edge was detected for the neem extract at 252 nm, while for MgO and MgO–neem NPs, it was 283 and 285 nm, respectively (Figure 4A). The literature indicates that the successful reduction of metal ions and the generation of metal oxide NPs were suggested by an apparent absorption edge in the 260–300-nm range when sodium hydroxide and plant extract were utilized in the NP manufacturing process.

To correlate the optical properties with the observed structural alterations of bioinspired MgO–neem NPs, Tauc’s Equation 8 was utilized to determine the optical band gap, E_g_ (Devanand et al., 2013):

αhv=Ahv−Egm2, (8)

where α is the absorption factor, h*ν* is the energy of the incident photon, and A and m are constants depending on the nature of the transition. Plotting the relationship between photon energy hν and (αhv)^2^ yielded the predicted optical band gap energy E_g_ of bioinspired MgO–neem NPs (Figure 4B). The band gap of bioinspired MgO–neem NPs (E_g_ = 4.701 eV) was greater than that of MgO NPs (E_g_ = 4.534 eV). This discrepancy was in line with the published findings and connected to the observed shift in the particle size of bioinspired MgO–neem NPs (Sharmila et al., 2019; Fatiqin et al., 2021).

#### 3.1.4 Fourier-transform infrared spectra

**FIGURE 5:**
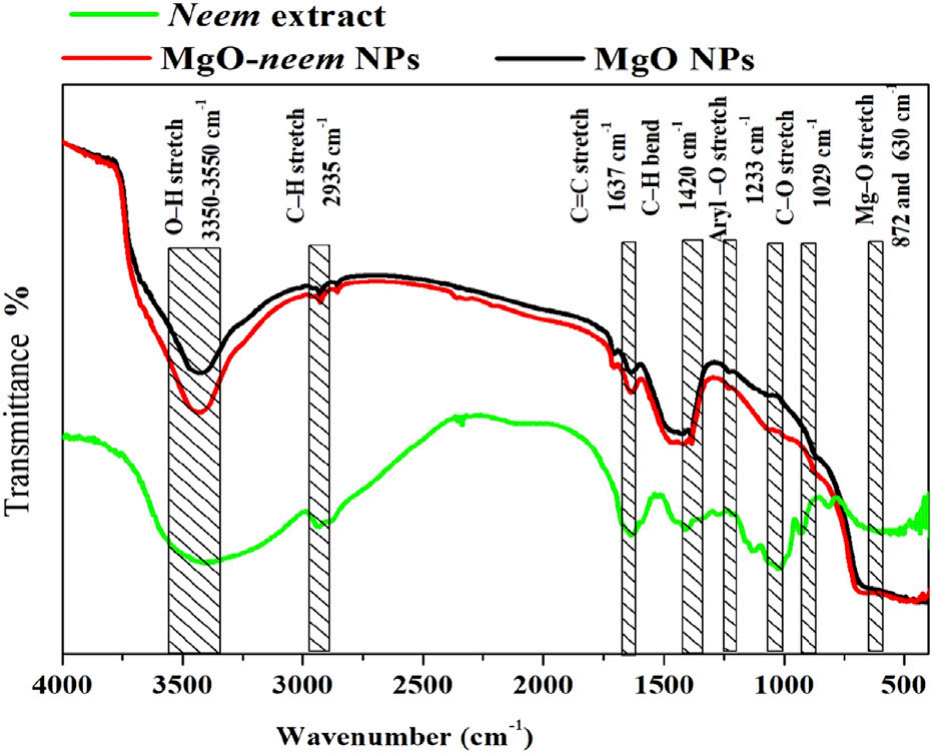
FTIR spectra of MgO and bioinspired MgO–neem NPs.

In the Fourier-transform infrared (FTIR) spectrum, three vibrational bands were identified as MgO NP fingerprints. The first band, which is related to the stretching vibration of magnesium oxide, is often observed between 600 and 880 cm^−1^. The second band, caused by the stretching of magnesium carboxylate, emerged between 1,600 and 1,640 cm ^−1^. Water is represented as moisture by the stretching hydroxyl (O-H) in the third broadband that appears between 3,350 and 3,550 cm^−1^. These bands were observed in the MgO and bioinspired MgO–neem NP spectra (Figure 5).

To investigate the role of biomolecules in the neem extract in the reduction of MgO NPs, the FTIR spectrum of the neem extract was analyzed. The broadband that was visible between 3,350 and 3,550 cm^−1^ was associated with the O-H vibration, which is indicative of amino acids and carbohydrates. Aromatic aldehyde stretching vibrations of C-H were observed at 2,935 cm^−1^. Conversely, at 1,630 cm^−1^, C=O stretching, which primarily originates from alkene compounds in proteins, was observed. The carbonyl group found in flavonoids was identified as the band observed at 1,590 cm^−1^. At approximately 1,400 cm^−1^, C-H bending vibration became apparent. Furthermore, the C-O stretching vibration originated in the aliphatic amine band at 1,029 cm^−1^.

Examining the FTIR spectra more closely revealed that the bioinspired MgO–neem NPs contained the functional groups (O-H, C-H, C=O, and C-O) that are associated with terpenoids, flavonoids, and proteins of the neem extract. Thus, the reduction and production of MgO NPs are mostly caused by these biomolecules. Research indicates that proteins’ C-N and C=O functional groups serve as capping agents to aid in the production of NPs (Chandrasekaran et al., 2024). Additionally, proteins’ amine linkages have a strong attraction to metals, which causes them to form a stabilized layer on the surface of the metal NPs.

#### 3.1.5 Stability measurement

The stability of NPs is one of the main challenges that hinder their potential applications. Measurement in conditions similar to *in vitro* or *in vivo* environments is an important yet hard feature of NP characterization. One of the critical factors is the stability and amount of aggregation of NPs under physiological conditions (e.g., plasma) or in various media relevant to biotechnological applications (e.g., culture medium). Several studies have demonstrated that the stability of NPs in various culture media can be significantly reduced depending on ionic and protein content, influencing NP characteristics and their functions in both *in vitro* and *in vivo* applications (Kadir et al., 2023; Proniewicz et al., 2024).

**FIGURE 6:**
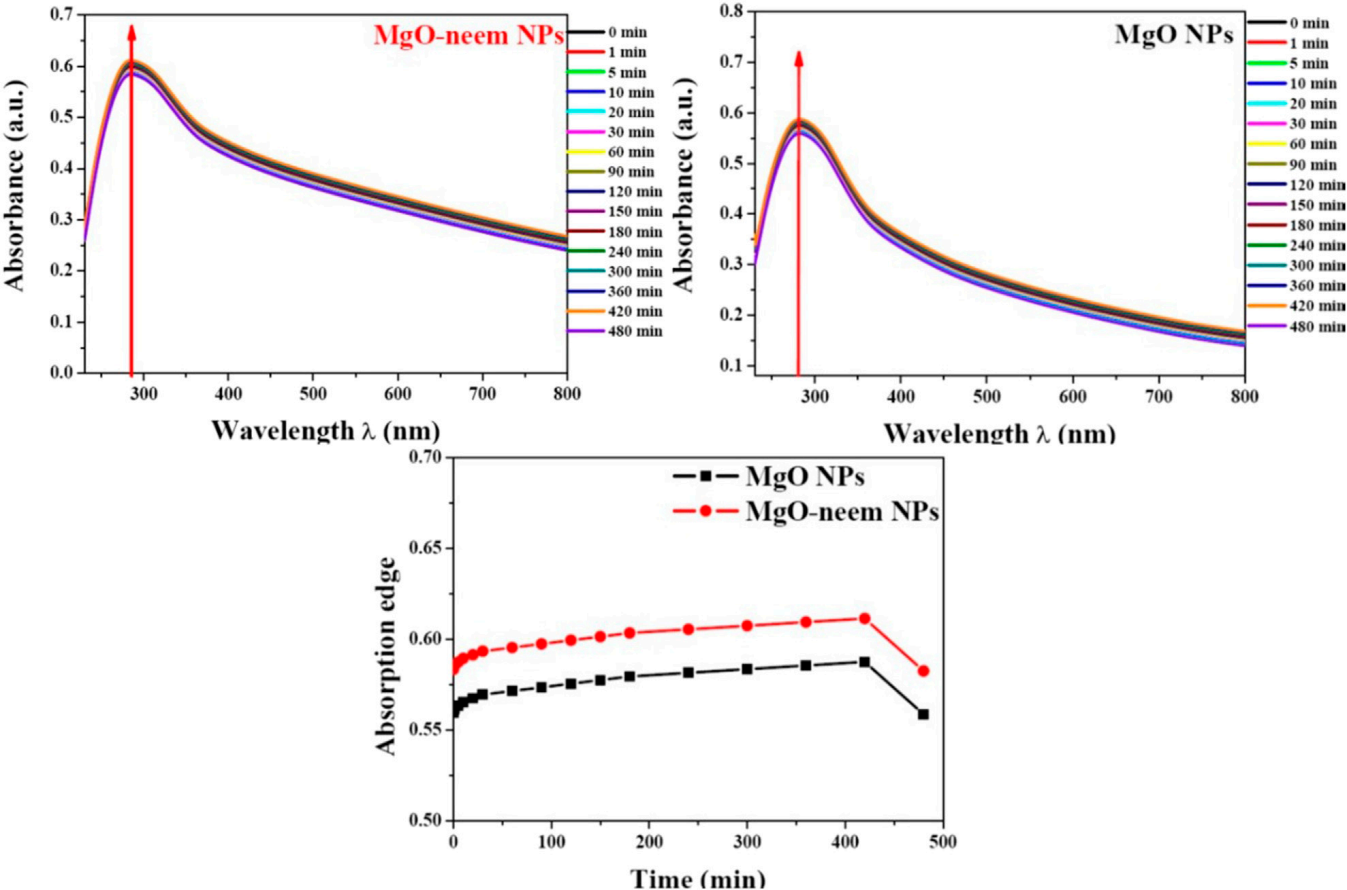
Stability of MgO and MgO–neem NPs in biological media. The top of the graph shows the UV spectra for each sample recorded at t time intervals (0–480 min). At the bottom, the dotted line represents the variation in the absorption edge with time.

For screening the stability of MgO NPs, UV spectroscopy was used. Both MgO NP samples were suspended in complete DMEM, and then UV spectra were recorded during different time intervals from 0 to 480 min (Figure 6). Both samples showed good stability during the measurement period (8 h), and there was no significant change in the absorption edge.

Thermogravimetric analysis (TGA) is a straightforward analytical method that calculates a material’s weight change (or gain) in relation to temperature. When materials are heated, they may lose weight by straightforward processes like drying or chemical reactions that release gases. The composition and structure of the material are closely linked to these thermal processes. To determine the material’s thermal behavior, TGA curves for any material may be divided into segments based on how the material’s weight loss changes with temperature.

**FIGURE 7:**
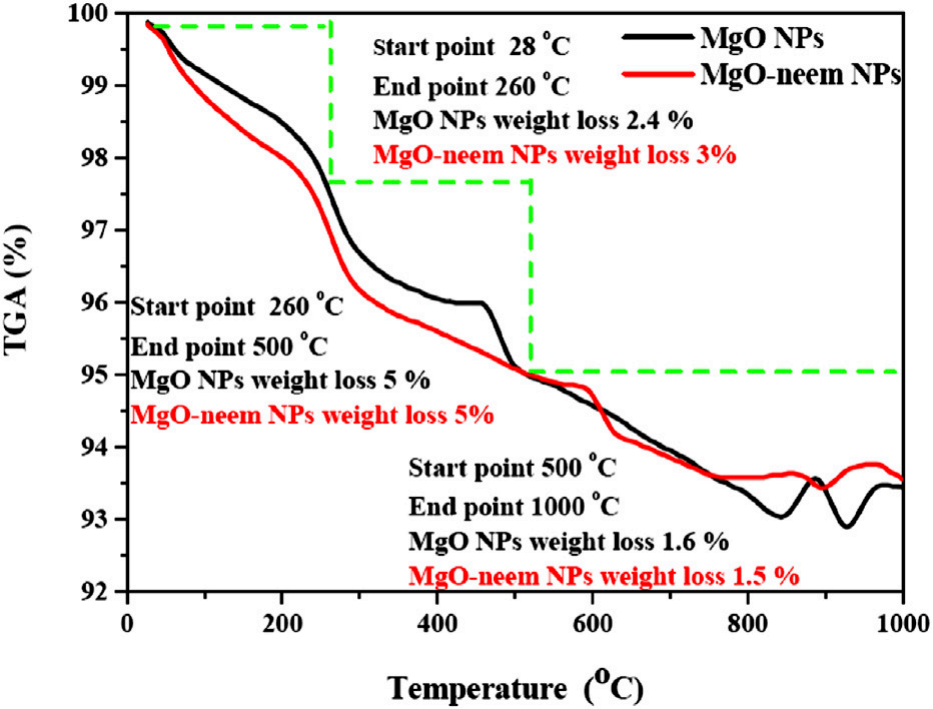
TGA curves of MgO and bioinspired MgO–neem NPs.

For MgO NPs, the weight loss was observed in three segments (Figure 7). The first segment starts from ambient temperature to 260^°^C with an approximate weight loss of 2.4% for MgO NPs and 3% for MgO–neem NPs. This weight loss was mainly due to the loss of absorbed water from moisture (Chandrasekaran et al., 2024; Hirphaye et al., 2023). The slight increase in the weight loss of bioinspired MgO–neem NPs due to the larger content of water was confirmed by the higher intensity of the stretching O-H band in the FTIR spectrum. The second segment, from 260 to 500^°^C, reflects the decomposition of organic molecules and the transition of MgO NPs (Poonguzhali et al., 2022; Leung et al., 2014). The weight loss in this segment was 5% for both MgO NPs and MgO–neem NPs, while the third segment lies between 500 and 1,000°C, in which the residue of organic molecules decomposes with smaller weight loss at approximately 1.6% and 1.5% for MgO NPs and MgO–neem NPs, respectively. This smaller weight loss indicates that both samples were stabilized in the crystalline phase above 500°C, which is in line with the reported thermal stability of MgO NPs (Leung et al., 2014; Sharmila and Selvaraj, 2024a).

### 3.2 Bioactivity of NPs

#### 3.2.1 Antioxidant activity (DPPH assay)

Creating free radicals is a cascade-like process; it starts with gaining or losing an electron, and this electron eventually hits another atom or molecule to create more free radicals. As a result, the reaction continues to produce an increasing number of these free radicals (Abdallah et al., 2019; Dobrucka, 2018). Antioxidant molecules are sufficiently stable to donate an electron to form a stable molecule and mitigate the damaging effects of free radicals. The literature suggests that the capacity to donate hydrogen is the reason behind the antioxidant activity of bioinspired MgO NPs. The production of an electron–hole pair on the surface of MgO NPs can significantly reduce H_2_O molecules, which can act as DPPH molecules’ scavengers. Nevertheless, because the plant extract contains phytochemicals, including phenolics and polyphenolic compounds, the green synthesis of MgO NPs may help modify the scavenging activity (Khan et al., 2020; Akshaykranth et al., 2021).

**FIGURE 8:**
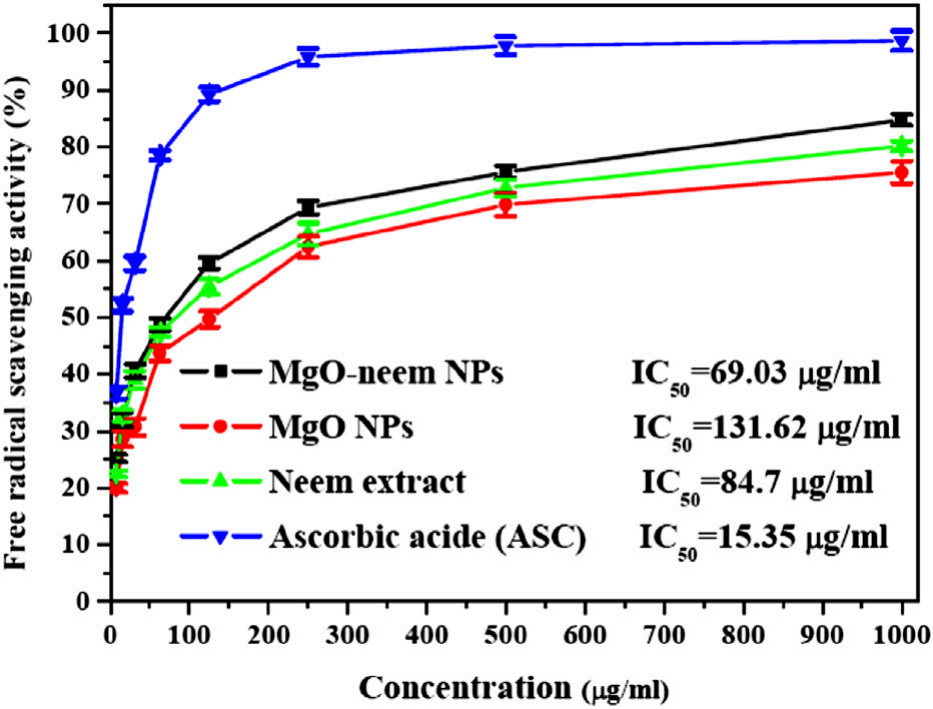
Free radical savaging activity of MgO NPs, MgO–neem NPs, neem extract, and ascorbic acid (ASC) as estimated using the DPPH assay.

The capability of the bioinspired MgO–neem NPs as a scavenger for DPPH radicals is compared to that of MgO NPs, neem extract, and ascorbic acid (ASC) as the standard and presented in Figure 8. A dose-dependent behavior was observed in the scavenging activity of all tested samples. The calculated IC_50_ value for bioinspired MgO–neem NPs was 69.03 μg/mL. The IC_50_ values for MgO NPs, neem extract, and ascorbic acid were 131.62 μg/mL, 84.7 μg/mL, and 15.35 μg/mL, respectively. The improved scavenging activity of the MgO–neem NPs can be mainly attributed to the presence of biomolecules from the neem extract, which enhance the NP efficacy. Previous studies on bioinspired MgO NPs from different biological sources have shown comparable DPPH scavenging activity (Table 3). These comparable scavenging values are bio-source-dependent, which indicates the importance of optimization of green MgO NP synthesis to achieve the desired bioactivity.

**TABLE 3:** Comparison of different bioactivities of bioinspired MgO NPs from different biological sources.

Measured activity	Bio-source	Findings	Reference
Antibacterial activity	*Painted spiral ginger*	Inhibition zone (mm) • *S. aureus* (5.50) • *B. subtilis* (10) • *E. coli* (12.5) • *S. paratyphi* (15)	Kainat et al. (2021)
	*Horseradish*	Inhibition zone (mm) • *S. aureus* (6.3) • *E. coli* (6)	Fatiqin et al. (2021)
	*Red pea*	Inhibition zone (mm) • *E. coli* (16.66) • *B. subtilis* (16) • *S. pyogenes* (15.66)	Abdullah and Mohammed (2021)
	*Shoeblack*	Inhibition zone (mm) • *P. aurigenosa* (19) • *P. vulgaris* (22) • *E. coli* (19)	Nadeem et al. (2021)
	*P. farcta*	Inhibition zone (mm) • *S. aureus* (18.21)	Rotti et al. (2023)
	*C. orientalis*	Inhibition zone (mm) • *K. pneumoniae* (14) • *P. aeruginosa* (18) • *S. aureus* (13) • *E. coli* (17) • *B. subtilis* (10)	Amina et al. (2020)
	*Azadirachta indica*	Inhibition zone (mm) • *P. aeruginosa* (33.5) • *E. coli* (28.7) • *S. aureus* (34.8)	Saied et al. (2021)
	*S. costus*	Inhibition zone (mm) • *E. coli* (15) • *P. aeruginosa* (16) • *S. aureus* (14) • *B. subtilis* (10)	Fouda et al. (2021)
	*A. terreus*	Inhibition zone (mm) • *C. albicans* (12.8) • *E. coli* (11.3) • *P. aeruginosa* (14.7) • *S. aureus* (11.3) • *B. subtilis* (13.3)	Pugazhendhi et al. (2019)
	*P. chrysogenum*	Inhibition zone (mm) • *S. aureus* (12) • *B. subtilis* (12.7) • *P. aeruginosa* (23.3) • *E. coli* (17.7) • *C. albicans* (14.7)	Sharma et al. (2022)
	*S. wightii*	Inhibition zone (mm) • *S. aureus* (9) • *P. aeruginosa* (8)	Ammulu et al. (2021)
Anticancer activity	*Painted spiral ginger*	• % Inhibition of Dalton’s lymphoma ascites: 52%	Kainat et al. (2021)
	*P. farcta*	• Inhibition zone for human breast cancer: 18 mm	Rotti et al. (2023)
	*S. costus*	• % Cytotoxicity of human breast cancer cells: 82%	Fouda et al. (2021)
	*S. wightii*	• % Apoptosis for human lung cancer cells: 79.5% % Cell viability: 20.5%	Ammulu et al. (2021)
Antioxidant activity	*Horseradish*	• IC_50_ value against DPPH: 290 μg/mL	Fatiqin et al. (2021)
	*Red pea*	• IC_50_ value against DPPH: 72.24 μg/mL	Abdullah and Mohammed (2021)
	*Shoeblack*	• % Scavenging DPPH: 69.2%	Nadeem et al. (2021)
	*C. orientalis*	• IC_50_ against DPPH: 22.65 μg/mL	Amina et al. (2020)
	*P. alba*	• % Scavenging DPPH: 69.2%	Thakur et al. (2022)
	*M. oleifera*	• IC_50_ value against DPPH: 290 μg/mL	Fatiqin et al. (2021)
	*P. marsupium*	• IC_50_ value against DPPH: 89.67 μg/mL	Gatou et al. (2024)
	*S. trilobatum*	• IC_50_ value against DPPH: 5.34 μg/mL	Narendhran et al. (2019)
Antifungal activity	Roots of *S. costus*	• Inhibition zone for *C. tropicalis* (20 mm) and *C. glabrata* (19 mm)	Fouda et al. (2021)
	*A. terreus*	• Inhibition zone for *C. albicans* (12.8 mm)	Pugazhendhi et al. (2019)
	*P. chrysogenum*	• Inhibition zone for *C. albicans* (14.7 mm)	Sharma et al. (2022)
Antidiabetic activity	*P. marsupium*	• IC_50_ value for alpha-amylase inhibition: 56.32 μg/mL • IC_50_ value for protein inhibition: 81.69 μg/mL	Gatou et al. (2024)
	*H. rosa-sinensis*	• IC_50_ value for alpha-amylase inhibition: 327 mg/mL • IC_50_ value for alpha-glucosidase inhibition: 400 mg/mL	Nadeem et al. (2021)

#### 3.2.2 Antibacterial activity

**FIGURE 9:**
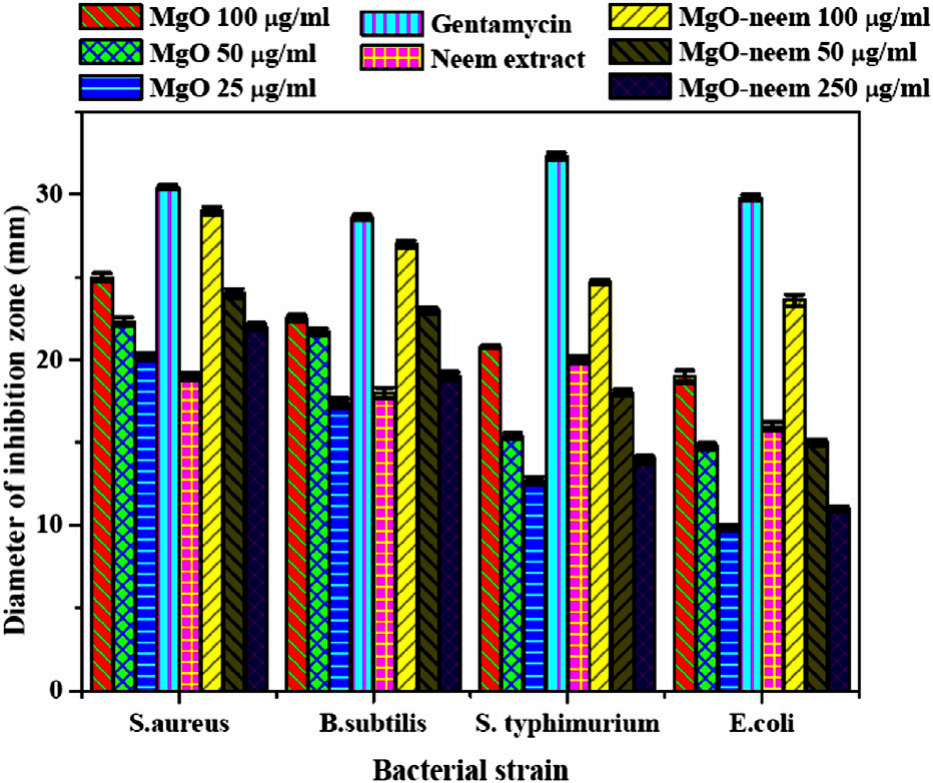
Inhibition zone (mm) of bioinspired MgO–neem NPs and MgO NPs; the represented values are the mean of three replicates. [**] *p* < 0.01 and [*] *p* < 0.05 compared to the test samples for all bacterial strains used in the study.

The MgO NPs’ antibacterial activity can be ascribed to two distinct mechanisms: the antimicrobial effect mediated by reactive oxygen species and the non-reactive species. MgO NPs create H_2_O_2_, which causes oxidative stress in the microbial system (Dobrucka, 2018; Nguyen et al., 2023). This leads to the production of reactive oxygen species (ROS), ultimately resulting in cell death. Furthermore, MgO NPs have been linked to cellular membrane disruption and contents leaking after physical contact. They may penetrate cells more quickly due to their smaller size, and they can interact with cells more extensively due to their larger surface area. Cell, protein, and DNA damage occur at higher MgO NP concentrations (Kumar et al., 2021; Breijyeh et al., 2020). For comparing the antibacterial activity of the investigated samples, a clearer zone of inhibition was measured in millimeters and is shown in Figure 9.

Bioinspired MgO–neem NPs recorded a significant activity for Gram-positive and Gram-negative bacterial strains in a concentration-dependent manner compared with MgO NPs and neem extract at all concentrations. This activity is close to the positive control Gentamicin, especially at the higher concentration (100 μg/mL). This behavior was attributed to the functionalization of MgO–neem NP surfaces with biomolecules from the neem extract. It is reported that the biological synthesis of NPs enhances the ROS generation ability, improving the antibacterial activity (Umaralikhan and Jamal, 2018; Suresh et al., 2018). The neem extract was also identified as a powerful antibacterial agent due to the high content of phenolic and flavonoid phytochemicals (Moorthy et al., 2015). The antibacterial action of bioinspired MgO NPs from different biological sources was reported against many pathogens (Table 3).

Moreover, when comparing the inhibition zones, Gram-negative bacterial strains (*S. typhimurium* and *E. coli*) exhibited smaller inhibition zones than the examined Gram-positive strains (*S. aureus* and *B. subtilis*) after treatment with all test samples. This behavior was reported for most Gram-negative bacterial strains, which was attributed to the stronger structure of Gram-negative bacteria than that of Gram-positive bacteria. Due to this structural variation, Gram-negative bacterial strains possess higher resistance to destruction than Gram-positive bacterial strains. However, bioinspired MgO–neem NPs showed a higher activity than MgO NPs at the three concentrations. These findings reflect the impact of the green synthesis of MgO NPs in treating different pathogens.

#### 3.2.3 Anticancer activity

**FIGURE 10:**
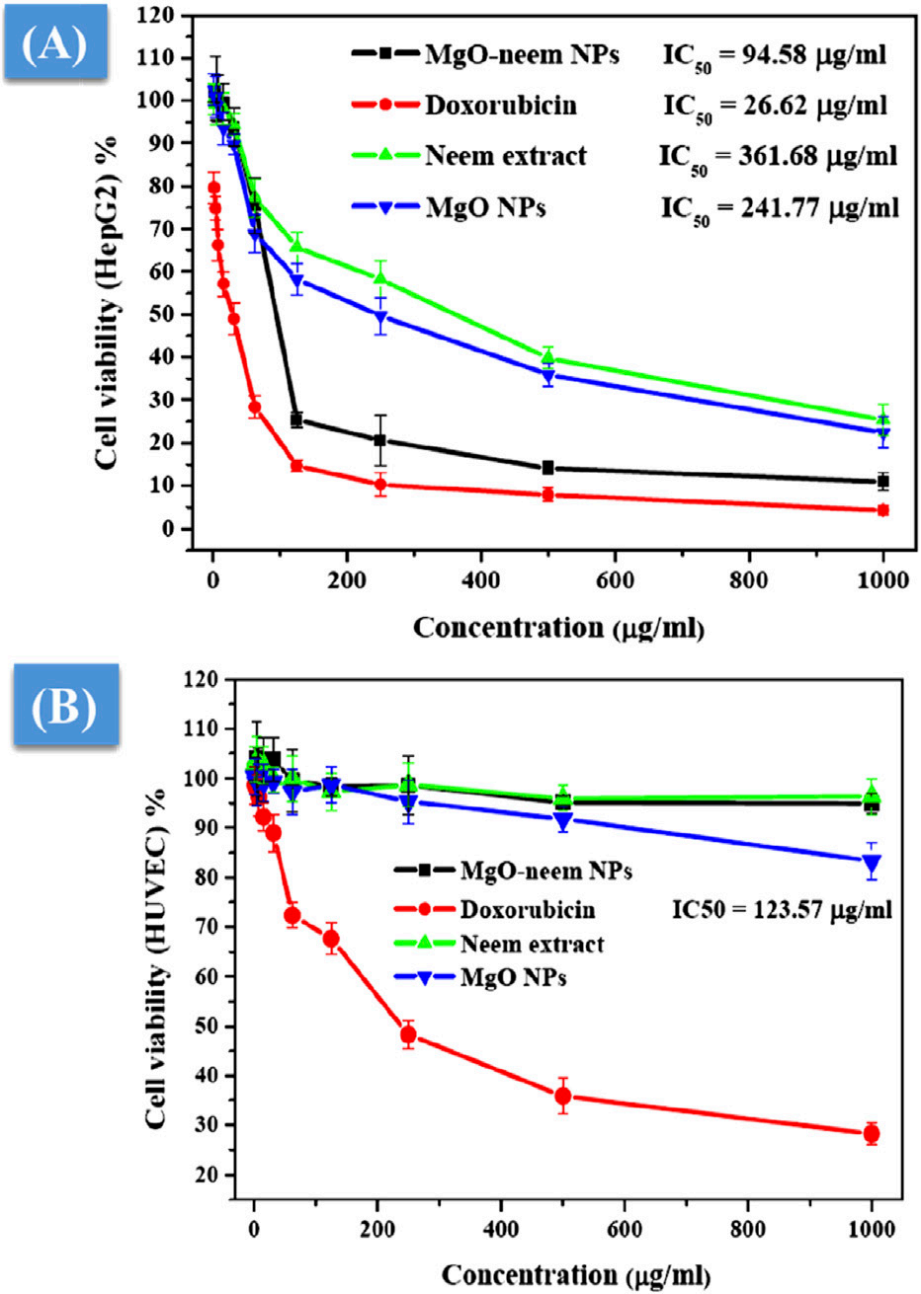
Cell viability after treatment with MgO–neem NPs, MgO NPs, neem extract, and doxorubicin (for 24 h) as evaluated from the MTT assay for the cancer cell line (HepG2) **(A)** and normal cell line (HUVECs) **(B)**.

As a distinct property, MgO NPs were characterized by the ability to generate ROS due to their high surface-to-volume ratio. The physicochemical properties of MgO NPs, such as size, shape, and surface reactivity, control the number of generated ROS. Upon entering the cellular membrane, these ROS cause oxidative stress, which, in turn, causes DNA damage, protein oxidation, mitochondrial malfunction, and, eventually, cell death (Velsankar et al., 2023; Majeed et al., 2018). The percentage of cell viability was determined for the tested samples by the colorimetric MTT assay as a function of mitochondrial activity and normalized to its respective control (Figures 10A, B). A significant concentration-dependent reduction in the cell viability of HepG2 cancer cells was observed after treatment with all samples. Bioinspired MgO–neem NPs showed distinct cancer cell-killing activity with an IC_50_ value of 94.58 μg/mL compared with doxorubicin (IC_50_ = 26.62 μg/mL), MgO NPs (IC_50_ = 241.77 μg/mL), and neem extract (IC_50_ = 361.68 μg/mL) (Figure 10A). There was no significant effect on the cell viability of normal cells except for cells treated with doxorubicin (IC_50_ = 123.57 μg/mL), (Figure 10B). These results demonstrate the high selectivity of MgO–neem NPs toward cancer cells, which is stimulated by their green synthesis and linked with antibacterial activity results (Fouda et al., 2021; Suba et al., 2022; Mangalampalli et al., 2019; Kessler et al., 2022; Mittag et al., 2019). Comparable findings were reported for bioinspired MgO NPs after exposure to different types of cancerous cells (Table 3).

#### 3.2.4 Oxidative stress assay

**FIGURE 11:**
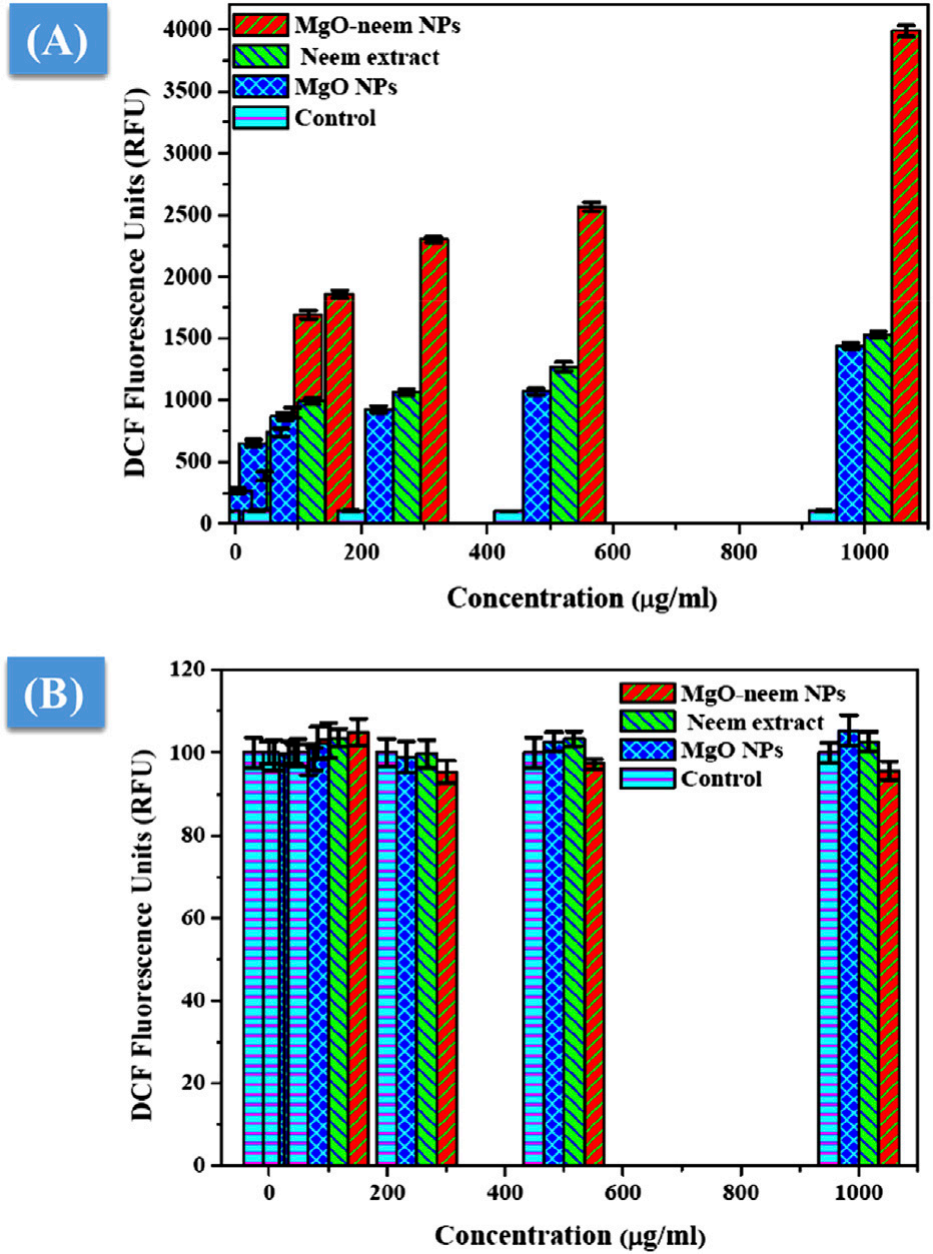
ROS level after exposure to MgO NPs, MgO–neem NPs, and neem extract for 24 h for the cancer cell line (HepG2) **(A)** and normal cell line (HUVECs) **(B)**.

MgO NPs cause ROS production under physiological conditions due to their large band gap, enabling them to donate hydrogen ions easily (Majeed et al., 2018; Mittag et al., 2019; Krishnamoorthy et al., 2012). This study assessed the generation of ROS following the exposure of two distinct cell lines to MgO NPs, MgO–neem NPs, and neem extract with variable concentrations for 24 h. Figure 11A shows an increase in ROS generation in HepG2 cancer cells that is dosage-dependent, which is consistent with the cytotoxicity and antibacterial findings, given that MgO–neem NPs exhibited increased toxicity against HepG2 cells compared with MgO NPs and neem extract. These findings show that exposure to MgO–neem NPs increases ROS levels, which, in turn, causes oxidative stress and cellular damage. In contrast, there was no notable increase in the level of ROS in either the untreated or normal HUVEC cells (Figure 11B). Many researchers have observed that MgO NPs have a stronger selectivity against cancer cells (Mittag et al., 2019; Poljsak et al., 2013; Joó et al., 2023). Since cancer cells have high rates of metabolism and proliferation, the presence of additional chemical and signaling components in MgO NPs increases their reactivity. These findings also aligned with the high scavenging activity of the obtained bioinspired MgO–neem NPs. In normal cell lines, the number of generated ROS is limited and can be eliminated by the antioxidant scavengers’ enzymes. Due to the high selectivity in cancer cells, the number of generated ROS is huge due to the high surface-to-volume ratio, which disturbs the balance between antioxidant activity and ROS and cannot be neutralized by the action of antioxidant scavengers’ enzymes (Gong et al., 2020; Alqahtani et al., 2020). These unique bioactivities of bioinspired MgO–neem NPs increase their potential as alternative agents in the biomedical field.

#### 3.2.5 Antidiabetic activity

**FIGURE 12:**
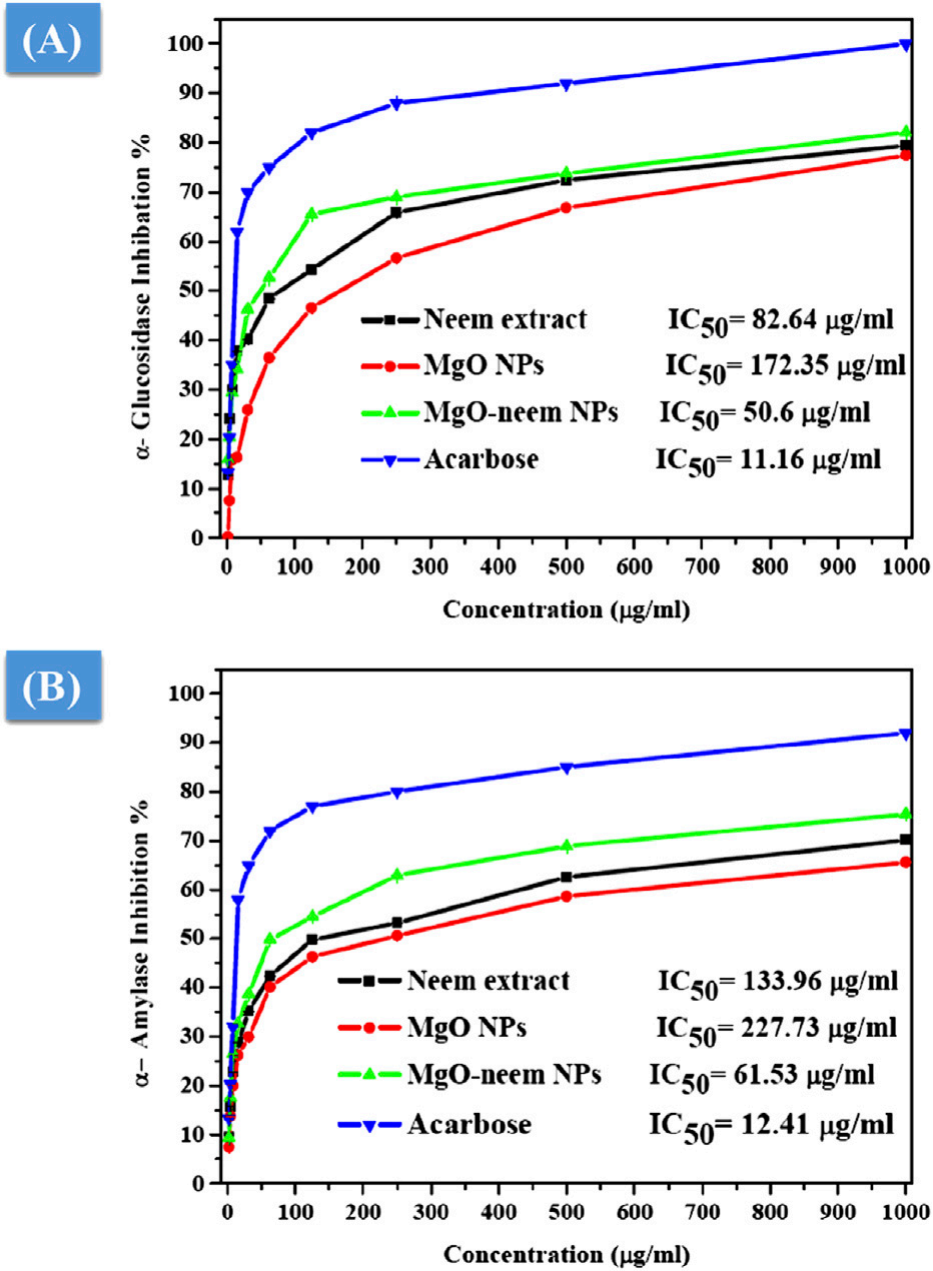
Antidiabetic activity of MgO NPs, MgO–neem NPs, neem extract, and acarbose at variable concentrations. The percentage of α-glucosidase inhibition *versus* test sample concentration **(A)** and the rate of α-amylase inhibition *versus* test sample concentration **(B)**.

The antidiabetic activity of MgO NPs is evaluated by the estimation of α-amylase and α-glucosidase inhibition percentages. It is reported that these two digestive enzymes break down carbohydrates into glucose. These two enzymes are crucial factors influencing the conversion of disaccharides and oligosaccharides into monosaccharides (Evans et al., 2002; Forman and Zhang, 2021). Therefore, the inhibition of these two enzymes is critical for the treatment of type-2 diabetes. The percentage of α-glucosidase inhibition was calculated spectrophotometrically using yeast α-glucosidase and pNPG after treatment with MgO NPs, MgO–neem NPs, neem extract, and acarbose with variable concentrations, as shown in Figure 12A.

Compared with acarbose as the standard with an IC_50_ value of 11.16 μg/mL, MgO–neem NPs showed concentration-dependent powerful inhibition with an IC_50_ value of 50.6 μg/mL. This behavior was linked to the high antioxidant activity of MgO–neem NPs. Moreover, neem extract and MgO NPs demonstrated comparable inhibition with IC_50_ values of 82.64 and 172.25 μg/mL, respectively. Similar behavior was observed for α-amylase inhibition percentage, and the test samples followed the same trend, starting with acarbose as the standard with an IC_50_ value of 12.41 μg/mL, MgO–neem NPs with an IC_50_ value of 61.53 μg/mL, neem extract with an IC_50_ value of 133.96 μg/mL, and MgO NPs with an IC_50_ value of 227.75 μg/mL (Figure 12B). It is reported that oxidative stress plays a critical role in the development of diabetes. An excess blood glucose level causes oxidative stress, which, in turn, causes glucose to auto-oxidize and free radicals to develop (Caturano et al., 2023; Chen et al., 2018; Dunkelberger and Song, 2010). Therefore, the treatment with MgO–neem NPs led to scavenging these free radicals, causing the inhibition of α-glucopyranoside and α-amylase enzymes. These results agreed with the report on bioinspired MgO NPs (Table 3).

#### 3.2.6 Anti-inflammatory activity

As a defense mechanism, inflammation involves blood vessels, immune cells, and molecular mediators. Inflammation has three main functions: it dissolves injured cells and tissues, starts tissue healing, and removes the source of cell harm. White blood cells in the human body use the process of inflammation to defend the body against harm or infection from external intruders like bacteria and viruses. A low level of inflammation may risk the organism’s life by allowing hazardous stimuli, like germs, to gradually destroy tissue (Ammulu et al., 2021; Sharmila and Selvaraj, 2024b), 102. An anti-inflammatory medication works to lessen inflammation. Regrettably, organisms’ tissues and organs may suffer more harm from unchecked inflammation. Therefore, finding natural sources of anti-inflammatory drugs is critical.

**FIGURE 13:**
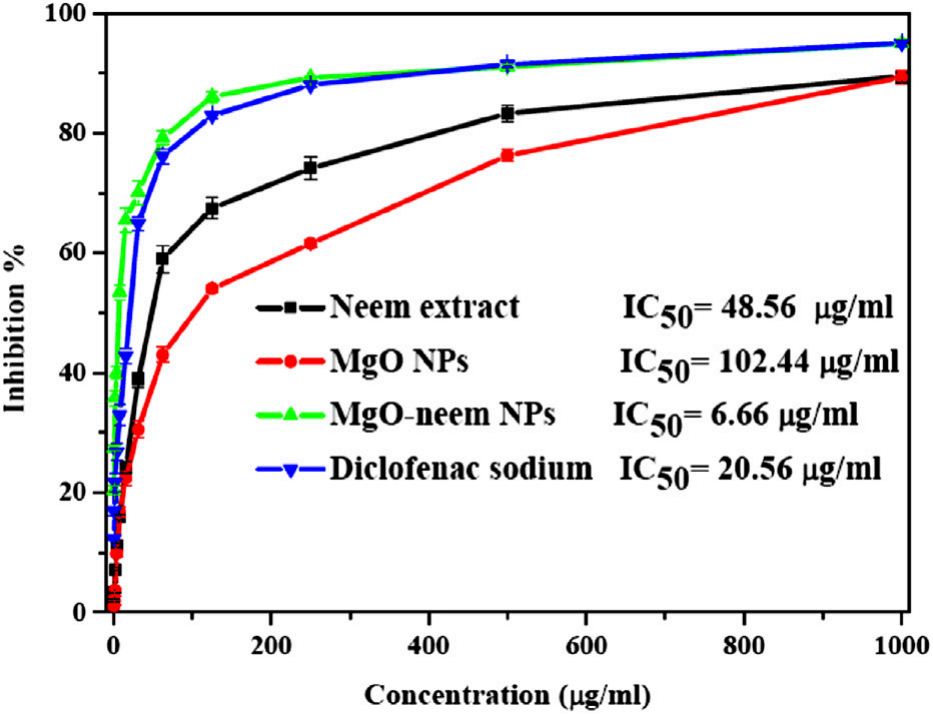
Protein denaturation inhibition percentage of MgO NPs, MgO–neem NPs, neem extract, and diclofenac sodium at variable concentrations.

A protein denaturation assay was used to assess the anti-inflammatory action of MgO NPs, and the degree of denaturation inhibition indicated high anti-inflammatory action. The percentage of inhibition of diclofenac sodium as standard, MgO NPs, bioinspired MgO–neem NPs, and neem extract was calculated and is shown in Figure 13. Significant inhibition was recorded for MgO–neem NPs with an IC_50_ value of 6.66 μg/mL compared with that of diclofenac sodium with an IC_50_ value of 20.56 μg/mL, while neem extract and MgO NPs showed less inhibition with IC_50_ values of 48.56 and 102.44 μg/mL, respectively. Traditionally, neem extract is used to treat inflammation. The primary cause of the enhanced anti-inflammatory properties of MgO–neem NPs is their phytochemical content, mainly the phenolic and flavonoid components. These phytochemicals enhance ROS generation, as we mentioned before, leading to the activation of pro-inflammatory cytokines and interleukins (Gatou et al., 2024; Sharmila and Selvaraj, 2024a).

## 4 Conclusion

Comprehensive studies were conducted to assess the effect of the neem extract on the physiochemical properties and bioactivity of MgO nanoparticles. For comparative purposes, MgO NPs were synthesized chemically by the chemical precipitation approach, using sodium hydroxide as a reducing gent, and biologically using the neem extract as a reducing gent. Structural investigations showed that the cubic face-centered structure of MgO NPs remained unchanged after green synthesis. The produced MgO NPs showed good stability in biological media for 8 h, with thermal stability up to 500°C. The neem extract’s biomolecules, which primarily modify the particle size and optical band gap, are responsible for the bioreduction of magnesium ions. These biomolecules also improve the ability of bioinspired MgO–neem NPs to scavenge free radicals, which, in turn, boost their antioxidant and antidiabetic activities through the inhibition of α-glucopyranoside and α-amylase enzymes. Furthermore, the bioinspired MgO–neem NPs’ enlarged optical band gap facilitates their easy donation of hydrogen ions, which affects the formation of ROS under physiological conditions. Therefore, the antibacterial, anticancer, and anti-inflammatory properties of bioinspired MgO–neem NPs are mostly attributable to these ROS. Consequently, bioinspired MgO–neem NPs demonstrated potent antibacterial activity against both Gram-positive and Gram-negative bacteria. Furthermore, cytotoxicity measurements verified the bioinspired MgO–neem NPs’ remarkable selectivity toward the hepatocellular cancer HepG2 cell line compared to normal human umbilical vein endothelial cells (HUVECs). This selectivity was corroborated by the fact that bioinspired MgO NPs generate more ROS in cancer cell lines than in normal cell lines. Moreover, an excess number of generated ROS can boost the anti-inflammatory capacity of bioinspired MgO NPs by activating the pro-inflammatory cytokines and interleukins that initiate the inflammatory process. Ultimately, the bioinspired MgO NPs show increased potential as a localized therapeutic agent for the treatment of many diseases due to their exceptional biocompatibility and antioxidant, antibacterial, anticancer, antidiabetic, and anti-inflammatory activities. However, further research is needed to investigate their pharmacokinetics as an alternative to existing natural medication on the market.

## Data Availability

The raw data supporting the conclusions of this article will be made available by the authors, without undue reservation.
